# Reception to Sir William MacCormac by the Chicago Medical Society

**Published:** 1883-11

**Authors:** 


### Society Reports.

---

ARTICLE VI.

FRATERNAL.

A reception to Sir William MacCormac, by the Chicago Medical Society, was the feature of a special meeting of this society at the Grand Pacific Hotel on the evening of the 26th ult., to greet this eminent Senior Surgeon of St. Thomas Hospital, Longon, who had obligingly remained in the city two days longer than he had previously decided upon doing.

Prof. S. J. Jones and Dr. R. G. Bogne escorted Sir William to the parlor that was well filled by the members, and presented to Prof. D. W. Graham, the president, who welcomed him in a most appropriate manner, and then introduced the distinguished visitor.

His response and preliminary remarks were most pleasantly complimentary to those present, after which he proceeded to give a very interesting address of much scientific value to the assembled physicians. This was of a full hour's duration, and his accent was so accrate that one would scarcely be reminded by it of the presence of a typical Englishman.

The first subject treated was that of plastic surgery, and he cited instances in his own practice treatment for deformities caused by burns; illustrations of this accident occurring about the hip, knee, breast, and cervical region were given.

Rhinoplasty was next dwelt upon, and cases were cited where he had been successful in transplanting skin from the arm and restoring the contour of the nose, as this prominent and useful organ is regarded as being quite indispensible to a regular and well outlined facial expression. The talented speaker subsequently dwelt quite minutely upon the practice of feeding the flaps in this and other surgical operations. Then the operation for extroversion of the bladder, and the various points illustrating this operation were made by drawing upon the blackboard with colred chalk as the lecturer proceeded. Sir William then passed somewhat hurriedly (for want of more time) to abdominal surgery. Operations here should be performed at two intervals, and this was especially true in "gastrostomy." First there should be performed (what he was pleased to term) an exploratory operation, to be followed in a few days or weeks, by an opening into the œsophagus or stomach, which could be done safely with proper precautions. Œsophagotomy, gastrotomy and gastrostomy and operations on the liver for the removal of hydatid cysts he had performed successfully with much relief and benefit to patients. Two cases of malignant disease of the stomach occurring in men, aged respectively 55 and 42, in his private practice were reported. Both patients had become greatly emaciated, anæmic and exhausted from inanition. In the oldest case the patient wanted a little more time to set his affairs in order; desired Sir William to make an incision in his stomach which was accordingly done, as above described, to the great benefit of the sufferer, who was not only relieved of the pain but derived great satisfaction in having the distress of hunger ameliorated by being fed through the opening in his side. The man became apparently at once in good health, visited the sea side, smoked a number of cigars a day, and thus lived 100 days is comparative comfort and luxury. The man expressed profound thanks for the relief afforded him; knew his time was approaching, but he had been relieved in whole, or for most of the time, from the great distress and constant acute pain.

In the other case spoken of, the patient had the same assemblage of symptoms, and was dying from the effects of the excruciating pain and debility dependent upon exhaustion, starvation, etc. The cancer had nearly closed the œsophagal opening into the stomach. He was operated upon in a similar manner as in the first case, and relief followed the surgical procedure. He was also fed through the opening by artificial means, and lived some six or seven months in this way.

Operations on the parenchyma of the kidney and body of these organs were alluded to for the various surgical maladies that may be seated there, or for traumatic lesions occurring to either organ. Operations for the different forms of hernia were dwelt upon as minutely as time would permit. The strangulated, inguinal, femoral and scrotal forms were each described, also the method of "dissecting out" the sac in which they may be enclosed, et cetera, et cetera. The treatment of gun-shot wounds, especially those in the late Egyptian war, received especial mention; that during the whole of it, though the troops suffered severely and endured much from want of water and from other causes of privation, there was not a single case of erysipelas, pyæmia or gangrene. The English Surgeon's work was the finest ever done in war, although many complaints in the public press had been made against them for their not using antiseptics and anæsthetics. This, however, has been proved to be untrue.

The antiseptic remedies, including Listerism, were methods largely employed by their surgeons, and regarded by them as surgical cleanliness. Regarding anæsthetics, he spoke of these as boonsand great blessings, discovered by American scientists; but thevalue of anæsthesia, in making operations painless is indescribable, and compared to the dire suffering a poor fellow formerly had to undergo in amputation, was beyond fitting words for him to express. Besides these great advantages, pyæmia and septicæmia are of much less frequency, or rare, when both an anæsthetic and antiseptic are used.

He closed his address by asking, What is surgery? After all, surgery is the work of the hands; but the surgeon's head and his heart he must use. He had met all these requisites in this wonderful city of Chicago, which to read about, how she arose from the ashes a few years since, like an Arabian Night tale. He had received from every one the greatest hospitality and kindness, which he regarded asdue to the fact that he was an English surgeon. All here and in Great Britain speak the same language and belong to a mother country. He desired to return thanks for the many kindnesses received. Sir William's remarks were greeted with sincere delight and loud applause.

President Graham announced that any who desired to speak, or to ask our their guest any question, were at liberty to do so.

Prof. N. S. Davis arose to say how little we appreciate what we do. The head plans, the hand is trained, and the heart pulsates, and pushes us on to devise operative appliances. Forty to fifty years ago, when he began practice, opium was given to relieve the pain, and antimony to relax the muscles, to prepare a patient for an operation, for then anæsthetics were unknown. He began his professional career with the idea of following surgery, but gradually drifted into a medical practice, because of locating in a somewhat rural district; but the progress of modern surgery in the past half century had been most wonderful. He responded fully and cordially to the sentiments of the fraternal and professional feeling between the profession in this and the mother country. They had a common language and common interests, and they all wished our honored guest many years of happiness, and many years to continue adding to the sum of human knowledge.

Prof. Charles T. Parkes expressed his great interest in the many points he had gained to-night from our distinguished guest, and spoke briefly upon the general subject of operative surgery, adducing instances in his own practice, especially one case of restoration of the ear. Ho was much pleased with the plan of treating strangulated hernia, by removing the sac entirely. He was also greatly pleased with the treatment called antiseptic surgery, as he thought these ideas were becoming lukewarm in the mind of the profession in this city. He closed with kind wishes and fraternal expressions for Sir William and the profession in England.

Prof. Moses Gunn said he had twenty-five years ago practiced the methods of feeding flaps spoken of by Sir William, and illustrated by the narration of a case of rhinoplasty to build up a chin that was missing. The dressings consisted of basilicon ointment and "*bandaging*" the parts. He had followed the remarks of the distinguished visitor with great interest, and heartily concurred in the remarks as to advantages of antiseptic surgery, which is surgical cleanliness, and Lister's method as the most advantageous, but he had abandoned the spray. However, the other details of antiseptic principles he followed out closely. The relations of the profession on both sides of the Atlantic were spoken of,and described in the same language. All have the same friendly feeling named by Prof. Davis, and it gave him the same pleasure to extend a courteous greeting.

Upon motion of Dr. C. G. Smith, that the thanks of the Society be tendered Sir William MacCormac, for his disseminating knowledge of some of the great surgical questions of the day, and also for the kind expressions of his good will to members of the profession in America, the meeting adjourned; which soon after, however, resolved itself into an informal "social and reception." A number of lady physicians were present. Besides these a number of scholarly gentlemen outside the profession were noticeable, who, with the members of the Society, were introduced, to say a few words and shake hands with the guest of the evening.

A number of regrets were received from those who were unavoidably absent.

Good-bys were spoken at a late hour with united regret on the part of "host and guest," mingled with much pleasure from all present in the experience of a profitable evening. L. H. M.

